# Patterns of Frailty in Older Adults: Comparing Results from Higher and Lower Income Countries Using the Survey of Health, Ageing and Retirement in Europe (SHARE) and the Study on Global AGEing and Adult Health (SAGE)

**DOI:** 10.1371/journal.pone.0075847

**Published:** 2013-10-29

**Authors:** Kenneth Harttgen, Paul Kowal, Holger Strulik, Somnath Chatterji, Sebastian Vollmer

**Affiliations:** 1 ETH Zürich, NADEL, Zürich, Switzerland; 2 World Health Organization, Geneva, Switzerland; 3 University of Göttingen, Department of Economics, Göttingen, Germany; 4 Harvard School of Public Health, Department of Global Health and Population, Boston, Massachusetts, United States of America; Federal University of Rio de Janeiro, Brazil

## Abstract

We use the method of deficit accumulation to describe prevalent and incident levels of frailty in community-dwelling older persons and compare prevalence rates in higher income countries in Europe, to prevalence rates in six lower income countries. Two multi-country data collection efforts, SHARE and SAGE, provide nationally representative samples of adults aged 50 years and older. Forty items were used to construct the frailty index in each data set. Our study shows that the level of frailty was distributed along the socioeconomic gradient in both higher and lower income countries such that those individuals with less education and income were more likely to be frail. Frailty increased with age and women were more likely to be frail in most countries. Across samples we find that the level of frailty was higher in the higher income countries than in the lower income countries.

## Introduction

Frailty is increasingly common as a result of population aging [1]–[4]. Assessing levels of frailty in higher versus lower income countries gives a unique perspective on health status at different stages of demographic transitions, and the effect of different policies on functioning and, in turn, on well-being over the life course. With almost all countries in the world faced with growing numbers of older persons, and more older persons in lower than higher income countries, a better understanding of levels of functioning and predictors of health care utilization will be necessary for planning purposes.

The important factors will be how to best maintain the health and functioning of an aging population, thereby preventing or postponing disease, disability and frailty. These mitigating factors are not only present at the individual level, but also in the supporting environment, for example in communities and social networks [5].

Frailty has significant impacts on individuals and society with increased risk of dependency, disability, hospitalization, institutional placement, and mortality [6], [7]. Numerous classifications and definitions of frailty are currently used in different clinical and research settings [8]. Although a common metric for measuring and assessing frailty would be a starting point, it is hard to establish [9]. Two of the more commonly used definitions are operationalized as: a physical phenotype [10]; and as a multi-domain count of health impairments [11]. The latter description of frailty, as a ‘multidimensional loss of reserves (energy, physical ability, cognition, health) that gives rise to vulnerability’, was used for this study [12], [13]. This approach accounts for deficits in many different health domains measured in the two multi-country studies used here: the Study on Health, Ageing and Retirement in Europe (SHARE) and the World Health Organization's (WHO) Study on global AGEing and adult health (SAGE).

This paper provides evidence on frailty in older adults across 14 higher income countries and six lower income countries, using a common set of variables to define frailty. The background aging characteristics of each of the countries in the study are presented in Table 1.

**Table 1:** Population totals and percentage of older adults for the World, SHARE countries and SAGE countries in 2010 and 2030 (projected).

	2010						2030 (projected)					
Country	Total, N*	Median Age	60+, N	(%)	65+, N	(%)	Total, N	Median Age	60+, N	(%)	65+, N	(%)
*World*	*6,895*	*29.2*	*759*	*(11.0)*	*524*	*(7.6)*	*8,321*	*34.1*	*1378*	*(16.6)*	*976*	*(11.7)*
SHARE countries												
Austria	8.4	41.8	1.9	(23.1)	1.5	(17.6)	8.6	47.0	2.8	(32.9)	2.2	(25.1)
Belgium	10.7	41.2	2.5	(23.4)	1.9	(17.4)	11.2	43.8	3.4	(29.8)	2.6	(23.3)
Czech Republic	10.5	39.4	2.3	(21.8)	1.6	(14.8)	10.8	45.7	3.0	(27.7)	2.3	(21.4)
Denmark	5.6	40.6	1.3	(23.3)	0.9	(16.5)	5.9	42.1	1.7	(28.9)	1.3	(22.3)
France	62.8	39.9	14.4	(23.0)	10.6	(16.8)	68.5	42.4	19.9	(29.1)	15.8	(23.1)
Germany	82.3	44.3	21.4	(26.0)	16.8	(20.4)	79.5	48.8	28.8	(36.2)	22.3	(28.0)
Greece	11.4	41.4	2.8	(24.3)	2.1	(18.6)	11.6	47.4	3.6	(30.7)	2.7	(23.3)
Ireland	4.5	34.7	0.7	(16.5)	0.5	(11.7)	5.3	39.8	1.2	(23.2)	0.9	(17.5)
Italy	60.6	43.2	16.0	(26.5)	12.3	(20.4)	60.9	49.7	20.9	(34.4)	16.1	(26.4)
Netherlands	16.6	40.7	3.6	(21.8)	2.5	(15.3)	17.3	44.3	5.4	(31.3)	4.2	(24.2)
Poland	38.3	38.0	7.4	(19.2)	5.2	(13.6)	37.8	44.9	10.2	(27.0)	8.2	(21.7)
Spain	46.1	40.1	10.3	(22.3)	7.8	(17.0)	50.0	48.0	15.3	(30.7)	11.6	(23.2)
Sweden	9.4	40.7	2.3	(24.9)	1.7	(18.2)	10.4	42.3	3.0	(28.8)	2.3	(22.6)
Switzerland	7.7	41.4	1.7	(22.8)	1.3	(16.7)	8.1	46.6	2.6	(32.3)	2.0	(24.7)
SAGE countries												
China	1,341	34.5	165.2	(12.3)	109.8	(8.2)	1,393	42.5	340	(24.4)	229	(16.5)
Ghana	24.4	20.5	1.4	(5.9)	0.9	(3.8)	36.5	24.1	2.9	(7.9)	1.9	(5.2)
India	1,224	25.1	92.7	(7.6)	60.3	(4.9)	1,523	31.2	188	(12.3)	126	(8.3)
Mexico	113	26.6	10.2	(9.0)	7.2	(6.3)	135	34.2	22.4	(16.6)	15.8	(11.7)
Russian Federation	143	37.9	25.5	(17.8)	18.3	(12.8)	136	43.3	33.4	(24.5)	26.1	(19.1)
South Africa	50.1	24.9	3.7	(7.4)	2.3	(4.6)	54.7	29.1	6.0	(11.0)	4.3	(7.8)

Note:

* N in millions (,000,000).

*Source: UN Population Division, 2011.*

## Data and Methods

### The study sample

The data for these analyses come from two multi-country surveys, SHARE which is supported by the European Commission (see www.share-project.org), and SAGE which is supported by the US National Institute on Aging (see www.who.int/healthinfo/systems/sage). SHARE includes more than 45,000 individuals aged 50-plus [14] from higher income countries. Currently, four waves of SHARE are publicly available and fieldwork has started for the fifth. We focus on the second wave of SHARE, because its timing coincides (more or less) with the first wave of SAGE. The second wave of SHARE was conducted in 2006–07 and included 14 countries: Austria, Belgium, the Czech Republic, Denmark, France, Germany, Greece, Italy, Ireland, the Netherlands, Poland, Spain, Sweden, and Switzerland. SHARE is representing about 12% of the world's population aged 50-plus in 2011 [15].

SAGE consists of nationally representative samples of older adults in six low and middle income countries: China, Ghana, India, Mexico, the Russian Federation and South Africa, accounting for 42% of the world's population aged 50-plus in 2011 [15]. SAGE includes questions about health and well-being, risk factors and chronic conditions, socio-economic status and work, social networks, and health care utilization. Standardized training and survey materials were used across the countries. The first (and to date only publicly available) full wave of SAGE was implemented between 2007 and 2010 and provides information on over 42,000 individuals aged 18-plus in total, including 34,124 individuals aged 50-plus. Both studies, SAGE and SHARE, are still ongoing.

### Study contents and variables

In addition to socio-economic variables, including education, assets, income, consumption and transfers data, both data sets include a large set of health information (self-reported health, health conditions, physical and cognitive functioning, health behaviors, and health care utilization), biomarkers (grip strength, body mass index (BMI), lung function, vision), and well-being variables (affective health, subjective well-being, life satisfaction).

Education levels in SAGE countries were mapped onto an international standard in order to improve comparability of the highest level of education achieved across the countries [16]. Income quintiles in SAGE countries were generated from the household ownership of durable goods, dwelling characteristics (type of floors, walls and cooking stove), and access to services such as improved water, sanitation and cooking fuel. Durable goods included number of chairs, tables or cars, and if, for example, the household has electricity, a television, landline or mobile phone, or washing machine. The results were recoded into dichotomous variables taking the value of 0 if not present, and 1 if present. The data set was then reshaped and country-specific “asset ladders” were generated. A Bayesian post-estimation (empirical Bayes) method was used to arrange households on the asset ladder, where the raw continuous income estimates were transformed in the final step into quintiles. This dichotomous hierarchical probit model has been described in detail elsewhere and used in other studies using similar data [17]–[19].

Grip strength was measured using Smedley's hand dynamometer, with two trials in each hand with the respondent in a seated position with the arm at a 90-degree angle and elbow close to the body. A single value was derived from taking the average of the best result in each hand. Body mass index was based on measured height and weight, and is equal to weight/height squared.

### Frailty index construction

The approach taken for this analysis is the deficits count approach to construct the frailty index. A frailty index is strongly associated with worsening health status and higher mortality risk when at least 30 deficits are included [20]–[22]. As more deficits are included, the precision of estimates increases, alternately, estimates become unstable when fewer than 10 deficits are included.

The actual number of deficits included in this frailty index was determined by the availability of the same variables in the second wave of SHARE and the first wave of SAGE, and by the following inclusion criteria: (1) deficits must be associated with the health status of the individual; (2) prevalence of deficits must increase with age; (3) deficits must not saturate too early; (4) when the accumulated deficits of an individual are considered as a whole, they should cover a range of systems (for example, not only impacting cognition).

A set of assumptions accompanied the inclusion criteria: a) a reasonable proportion of the population would have the characteristic; b) low levels of missing data - not more than 25% missing for a given variable; and, c) variables were an expression of a health deficit [20].

Thirty-nine variables from SHARE and 40 variables from the SAGE dataset were used to generate the frailty index. These variables included difficulties in functioning (like loss of mobility), limitations in activities of daily living (ADLs) (such as needing help with bathing), and diseases (such as depression, stroke, diabetes). The underlying assumption is that increasing deficits contribute to a higher likelihood of frailty in an individual [23]. Information about variable selection and deficit thresholds for constructing the frailty index were taken from published literature [20], [24]. Table 2 shows the cut-offs of the selected variables and differences in constructing the frailty index for both SAGE and SHARE. Previous studies have indicated that frailty indices need not be based on the same items to yield similar results when taking the tallying deficits approach [25].

**Table 2:** List of variables included in the frailty index and coding criteria by domain and study.

	SHARE	SAGE
Topic/variable	Response categories and cut-points	
**General health (1):** Self-rated health	**Very good = 0, Good = 0.25, Moderate = 0.50, Bad = 0.75, Very bad = 1**	
**Medically diagnosed conditions (9)**	**1 = yes, 0 = no**	
Arthritis	√	√
Asthma	√	√
Cataracts	√	√
Chronic Obstructive Pulmonary Disease	√	√
Depression	√	√
Diabetes	√	√
Hypertension	√	√
	*Parkinson's Disease*	*Angina*
Stroke	√	√
**Medical symptoms (3 (SHARE) or 4 (SAGE)). In the last 30 days how much…**	**None = 0, Mild = 0.25, Moderate = 0.50, Severe = 0.75, Extreme/cannot = 1**	
bodily aches or pains did you have?	*NA*	*√*
of a problem did you have with sleeping?	√	√
difficulty did you have in seeing (person or object) across the road?	√	√
difficulty did you have in seeing an object at arm's length?	√	√
**Functional activities assessment (13). In the last 30 days how much difficulty did you have with…**	**1 = yes, 0 = no**	**None = 0, Mild = 0.25, Moderate = 0.50 Severe = 0.75, Extreme/cannot = 1**
sitting for long periods	√	√
walking 100 meters	√	√
standing up from sitting down	√	√
standing for long periods	√	√
climbing one flight of stairs without resting	√	√
stooping, kneeling or crouching	√	√
picking up things with fingers	√	√
extending arms above shoulders	√	√
concentrating for 10 minutes	√	√
walking long distance (1 km)	√	√
carrying things	√	√
getting out of your home	√	√
enjoy what you are doing	√	√
**Activities of daily living (10). In the last 30 days how much difficulty did you have with…**	**1 = yes, 0 = no**	**None = 0, Mild = 0.25, Moderate = 0.50, Severe = 0.75, Extreme/cannot = 1**
taking care your of household responsibilities	√	√
joining community activities	√	√
bathing/washing	√	√
dressing	√	√
day-to-day work	√	√
moving around inside home	√	√
eating	√	√
getting up from lying down	√	√
getting to and using the toilet	√	√
getting where you want to go	(using a map outside the house)	(using private or public transport, if needed)
**BMI (1)**: Weight/(Height in meters)∧2	BMI≥18.5 - <25 = **0 (Normal)**	
	BMI ≥25 - <30 = **0.5 (Overweight)**	
	BMI <18.5 = **1 (Underweight)**	
	BMI ≥30 = **1 (Obese)**	
**Grip strength (1)**: Grip (in kg), (Left+Right hand)/2	(Male and 0<BMI< = 24 and grip< = 29)	
	or	
	(Male and 24<BMI< = 26 and grip< = 30)	
	or	
	(Male and 26<BMI< = 28 and grip< = 30)	
	or	
	(Male and 28<BMI< = 40 and grip< = 32)	
	or	
	(Female and 0<BMI< = 23 and grip< = 17)	
	or	
	(Female and 23<BMI< = 26 and grip< = 17.3)	
	or	
	(Female and 26<BMI< = 29 and grip< = 18)	
	or	
	(Female and 29<BMI< = 40 and grip< = 21)	
	= **1 (weak grip)**	
**Timed walk at usual pace (1)**	(≤0.4 m/sec) = **0 (Normal)**	
	(>0.4 m/sec) = **1 (Slow)**	
	Time (sec) over 20 feet (6 meters)	Time (sec) over 4 meters

The variables included were binary, ordinal or continuous. Binary variables were coded as 1 being a deficit and 0 as non-deficit. For continuous variables, additional categories between 0 (no deficit) and 1 (maximal deficit) were included. For example, self-rated health can be very good = 0, good = 0.25, moderate = 0.50, bad = 0.75, very bad = 1 allowing for a more flexible deficit threshold. Setting cut-offs for the ordinal and continuous variables (walking speed, BMI, grip strength, mobility problems) followed an approach based on published literature. Cut-offs were defined based on characteristics of the distribution of the deficits [20].

The frailty index was then calculated as the proportion of the number of deficits for an individual to the total number of deficits. For example, a respondent with reported cancer, hypertension, trouble sleeping and slow walking speed has a frailty index score of 4/40 (0.10). In this case, frailty could be a pre-disabled state, so an individual could be frail but without any limitations in ADLs; or frail persons could have comorbidity and disability (i.e., with limitations in ADLs).

In addition, besides the differences in some indicators, the data sets also differ in the cut-off point for some indicators. For functional activities and activities of daily living, the SHARE data only provide information whether difficulties exist ( = 1) or not ( = 0), whereas scalable response categories are available and were used for these variables in the construction of the frailty index for SAGE (for example, none = 0, mild = 0.25, moderate = 0.50, severe = 0.75, extreme = 1).

To make the results comparable across countries with different population structures, the frailty index is weighted using the standard population distribution based on the WHO World Standard [26].

### Statistical analysis

In a regression analysis we collapse the individual level data and calculate the average of the frailty index by country, sex and age. We apply a log transformation to the average age-specific frailty index and then estimate a simple linear model (OLS) of the average age-specific frailty index and age for every country and by sex. The log transformation is justified because we expect the age-specific frailty index to increase exponentially with age. We only consider ages between 50 and 85, because for older ages the country and sex specific samples sizes are too small to calculate the average age-specific frailty index with sufficient accuracy.

## Results

**Figure 1:**
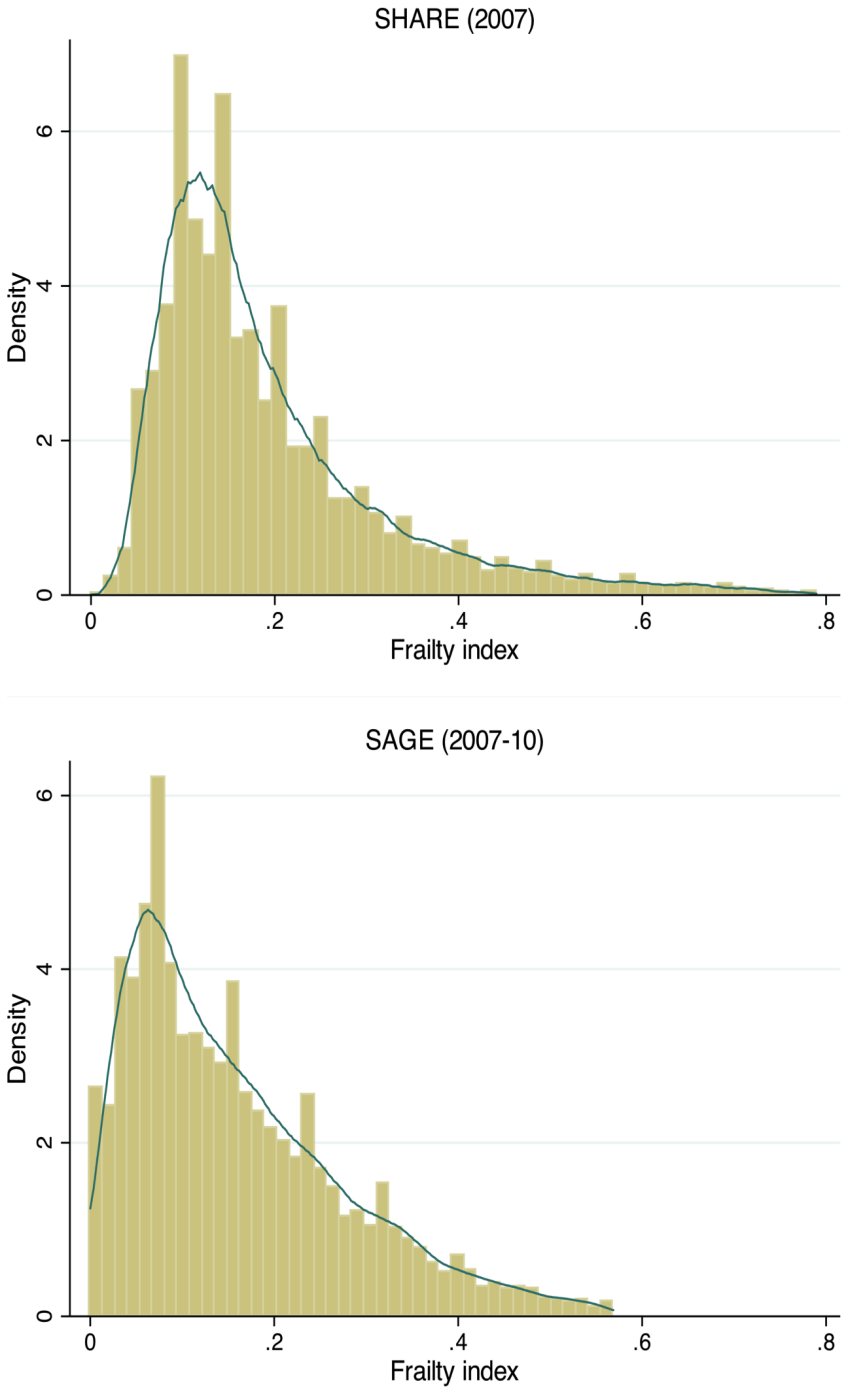
Distribution of the frailty index by study. Note: Histogram and kernel density estimate of frailty index for SHARE (2007) and SAGE (2007–10) studies. Individual level observations pooled across countries.

The results are based on weighted samples of persons aged 50-plus of N = 161,542 from SHARE 2007, and N = 18,566 from SAGE 2007–10 (see Tables 3 and 4). The shape of the density plots for SAGE and SHARE (Figure 1) looks rather similar, but with a slight left shift and lower peak in the case of SAGE.

**Table 3:** Mean and standard deviation of the frailty index in SHARE countries by sex, age, education and income.

Share 2007		Switzer-land	Denmark	Ireland	Sweden	Nether-lands	Greece	Germany	France	Czech Republic	Belgium	Austria	Italy	Spain	Poland
All	Mean	0.14	0.15	0.15	0.16	0.16	0.16	0.17	0.17	0.17	0.18	0.19	0.20	0.20	0.24
	*SD*	0.08	0.10	0.11	0.09	0.10	0.10	0.10	0.10	0.11	0.11	0.11	0.12	0.13	0.14
	N	7,125	12,675	1,107	13,555	13,075	15,405	12,640	14,351	13,795	15,450	6,620	14,635	10,910	12,145
**By Sex**															
Male	Mean	0.12	0.13	0.13	0.14	0.14	0.13	0.15	0.14	0.15	0.16	0.16	0.17	0.16	0.21
	*SD*	0.07	0.09	0.09	0.09	0.08	0.09	0.10	0.09	0.10	0.09	0.10	0.11	0.11	0.14
Female	Mean	0.15	0.16	0.17	0.17	0.18	0.18	0.18	0.19	0.19	0.20	0.20	0.22	0.23	0.26
	*SD*	0.08	0.10	0.12	0.10	0.10	0.11	0.11	0.11	0.11	0.11	0.12	0.13	0.13	0.14
**By Age**															
(50–54)	Mean	0.12	0.13	0.13	0.13	0.14	0.12	0.13	0.14	0.13	0.16	0.15	0.14	0.14	0.18
	*SD*	0.06	0.09	0.09	0.08	0.09	0.07	0.08	0.09	0.08	0.10	0.08	0.08	0.08	0.10
(55–59)	Mean	0.13	0.14	0.12	0.15	0.15	0.13	0.15	0.16	0.16	0.17	0.17	0.17	0.17	0.21
	*SD*	0.08	0.09	0.08	0.09	0.09	0.07	0.09	0.09	0.10	0.10	0.11	0.09	0.10	0.11
(60–64)	Mean	0.14	0.14	0.17	0.15	0.16	0.16	0.16	0.16	0.16	0.18	0.18	0.19	0.20	0.25
	*SD*	0.07	0.08	0.13	0.09	0.09	0.09	0.09	0.09	0.09	0.10	0.09	0.10	0.11	0.14
(65–69)	Mean	0.15	0.16	0.16	0.15	0.17	0.18	0.18	0.18	0.19	0.20	0.19	0.21	0.21	0.28
	*SD*	0.08	0.10	0.11	0.08	0.10	0.10	0.10	0.09	0.11	0.10	0.11	0.11	0.11	0.15
(70–74)	Mean	0.16	0.17	0.19	0.17	0.18	0.22	0.21	0.20	0.22	0.21	0.21	0.25	0.24	0.31
	*SD*	0.11	0.10	0.12	0.09	0.11	0.12	0.12	0.11	0.12	0.11	0.12	0.15	0.14	0.16
(75–79)	Mean	0.16	0.20	0.19	0.19	0.18	0.25	0.22	0.23	0.23	0.22	0.22	0.28	0.28	0.36
	*SD*	0.09	0.14	0.13	0.11	0.12	0.14	0.13	0.13	0.14	0.13	0.13	0.18	0.15	0.18
(80–84)	Mean	0.19	0.22	0.25	0.21	0.20	0.31	0.29	0.26	0.30	0.28	0.29	0.31	0.33	0.39
	*SD*	0.11	0.15	0.15	0.14	0.12	0.16	0.16	0.16	0.16	0.16	0.17	0.18	0.18	0.19
(85–89)	Mean	0.21	0.29	0.27	0.26	0.28	0.35	0.37	0.33	0.33	0.28	0.35	0.41	0.38	0.45
	*SD*	0.12	0.18	0.18	0.16	0.16	0.17	0.20	0.17	0.17	0.16	0.19	0.20	0.20	0.20
(90+)	Mean	0.29	0.30	0.33	0.37	0.29	0.39	0.41	0.40	0.35	0.41	0.33	0.44	0.44	0.47
	*SD*	0.18	0.17	0.23	0.18	0.17	0.16	0.17	0.15	0.23	0.17	0.15	0.22	0.17	0.17
**By Level of Education**															
Primary	Mean	0.16	0.18	.	0.16	0.16	0.17	0.20	0.18	0.19	0.20	0.23	0.20	0.19	0.29
	*SD*	0.09	0.11	.	0.10	0.11	0.11	0.13	0.12	0.12	0.13	0.13	0.12	0.13	0.16
Secondary	Mean	0.12	0.14	.	0.15	0.14	0.13	0.15	0.15	0.15	0.16	0.17	0.14	0.13	0.21
	*SD*	0.07	0.10	.	0.10	0.09	0.09	0.10	0.09	0.10	0.12	0.11	0.08	0.08	0.12
Higher	Mean	0.12	0.12	.	0.11	0.14	0.11	0.14	0.12	0.13	0.14	0.12	0.11	0.13	0.18
	*SD*	0.08	0.08	.	0.06	0.09	0.07	0.10	0.08	0.08	0.08	0.05	0.07	0.08	0.11
**By Income Quintile**															
Lowest	Mean	0.13	0.19	.	0.17	0.18	0.13	0.18	0.19	0.19	0.17	0.24	0.19	0.21	0.26
	*SD*	0.08	0.13	.	0.12	0.12	0.08	0.13	0.12	0.12	0.12	0.14	0.12	0.14	0.15
Second	Mean	0.15	0.14	.	0.16	0.16	0.20	0.16	0.17	0.19	0.21	0.21	0.20	0.22	0.27
	*SD*	0.10	0.09	.	0.10	0.11	0.12	0.11	0.12	0.12	0.14	0.13	0.13	0.14	0.16
Middle	Mean	0.13	0.12	.	0.14	0.14	0.17	0.15	0.16	0.18	0.19	0.19	0.18	0.17	0.25
	*SD*	0.07	0.08	.	0.08	0.10	0.11	0.10	0.10	0.11	0.14	0.11	0.11	0.12	0.14
Fourth	Mean	0.11	0.11	.	0.13	0.14	0.13	0.14	0.13	0.15	0.15	0.14	0.16	0.15	0.23
	*SD*	0.06	0.07	.	0.08	0.08	0.10	0.10	0.09	0.09	0.09	0.08	0.10	0.10	0.14
Highest	Mean	0.11	0.11	.	0.12	0.13	0.13	0.13	0.13	0.13	0.14	0.13	0.13	0.14	0.19
	*SD*	0.06	0.07	.	0.09	0.08	0.09	0.09	0.08	0.08	0.08	0.08	0.09	0.09	0.11

Note: N is the sample size of the respective survey. Estimates are weighted using the standard population distribution based on the WHO World Standard. For educational attainment we used a standard coding based on the 1997 International Standard Classification of Education ISCED-97 (UNESCO [16]). Income quintiles are calculated based on annual total household income. Countries sorted in increasing order by the mean of the frailty index.

**Table 4:** Mean and standard deviation of the frailty index in SAGE countries by sex, age, education and wealth.

Sage 2007–2010		China	Mexico	Ghana	South Africa	India	Russian Federation
All	Mean	0.10	0.13	0.14	0.15	0.15	0.16
	*SD*	0.08	0.12	0.11	0.12	0.11	0.11
	N	1,430	2,204	3,479	2,244	8,093	931
**By Sex**							
Male	Mean	0.08	0.11	0.12	0.13	0.14	0.13
	*SD*	0.07	0.11	0.10	0.11	0.11	0.10
Female	Mean	0.11	0.15	0.16	0.16	0.15	0.17
	*SD*	0.08	0.12	0.11	0.12	0.10	0.12
**By Age**							
(50–44)	Mean	0.07	0.08	0.10	0.12	0.12	0.10
	*SD*	0.06	0.08	0.09	0.11	0.09	0.08
(55–59)	Mean	0.09	0.13	0.14	0.16	0.18	0.16
	*SD*	0.07	0.11	0.10	0.12	0.10	0.10
(60–64)	Mean	0.11	0.16	0.16	0.16	0.20	0.19
	*SD*	0.08	0.11	0.10	0.12	0.11	0.11
(65–69)	Mean	0.13	0.15	0.19	0.18	0.23	0.22
	*SD*	0.10	0.12	0.11	0.13	0.13	0.12
(70–74)	Mean	0.14	0.19	0.22	0.20	0.26	0.25
	*SD*	0.09	0.13	0.11	0.13	0.12	0.12
(75–79)	Mean	0.18	0.22	0.24	0.20	0.28	0.28
	*SD*	0.10	0.14	0.12	0.13	0.11	0.12
(80–84)	Mean	0.18	0.25	0.27	0.21	0.31	0.33
	*SD*	0.08	0.14	0.13	0.14	0.13	0.13
(85–89)	Mean	0.21	0.29	0.27	0.20	0.33	0.31
	*SD*	0.14	0.15	0.12	0.13	0.13	0.15
(90+)	Mean	0.35	0.37	0.33	0.33	0.35	0.23
	*SD*	0.15	0.10	0.13	0.18	0.11	0.27
**By Level of Education**							
No education	Mean	0.11	0.16	0.16	0.17	0.18	0.25
	*SD*	0.08	0.12	0.11	0.13	0.11	0.15
Primary	Mean	0.10	0.13	0.11	0.15	0.14	0.29
	*SD*	0.08	0.11	0.10	0.11	0.10	0.14
Secondary	Mean	0.08	0.09	0.12	0.13	0.12	0.20
	*SD*	0.07	0.09	0.11	0.11	0.09	0.13
Higher	Mean	0.06	0.09	0.11	0.10	0.10	0.14
	*SD*	0.05	0.10	0.09	0.11	0.08	0.10
**By Income Quintile**							
Lowest	Mean	0.13	0.15	0.14	0.14	0.16	0.14
	*SD*	0.09	0.12	0.11	0.11	0.11	0.12
Second	Mean	0.11	0.15	0.14	0.16	0.15	0.20
	*SD*	0.09	0.13	0.11	0.12	0.11	0.13
Middle	Mean	0.08	0.13	0.14	0.15	0.15	0.16
	*SD*	0.06	0.12	0.11	0.12	0.10	0.11
Fourth	Mean	0.08	0.12	0.14	0.15	0.14	0.14
	*SD*	0.07	0.10	0.11	0.12	0.10	0.10
Highest	Mean	0.07	0.12	0.13	0.13	0.14	0.14
	*SD*	0.06	0.11	0.10	0.12	0.10	0.10

Note: Estimates are weighted using the standard population distribution based on the WHO World Standard. For educational attainment we used a standard coding based on the 1997 International Standard Classification of Education ISCED-97 (UNESCO [16]). Income quintiles in SAGE countries were generated from the household ownership of durable goods, dwelling characteristics and access to services. Countries sorted in increasing order by the mean of the frailty index.

**Figure 2:**
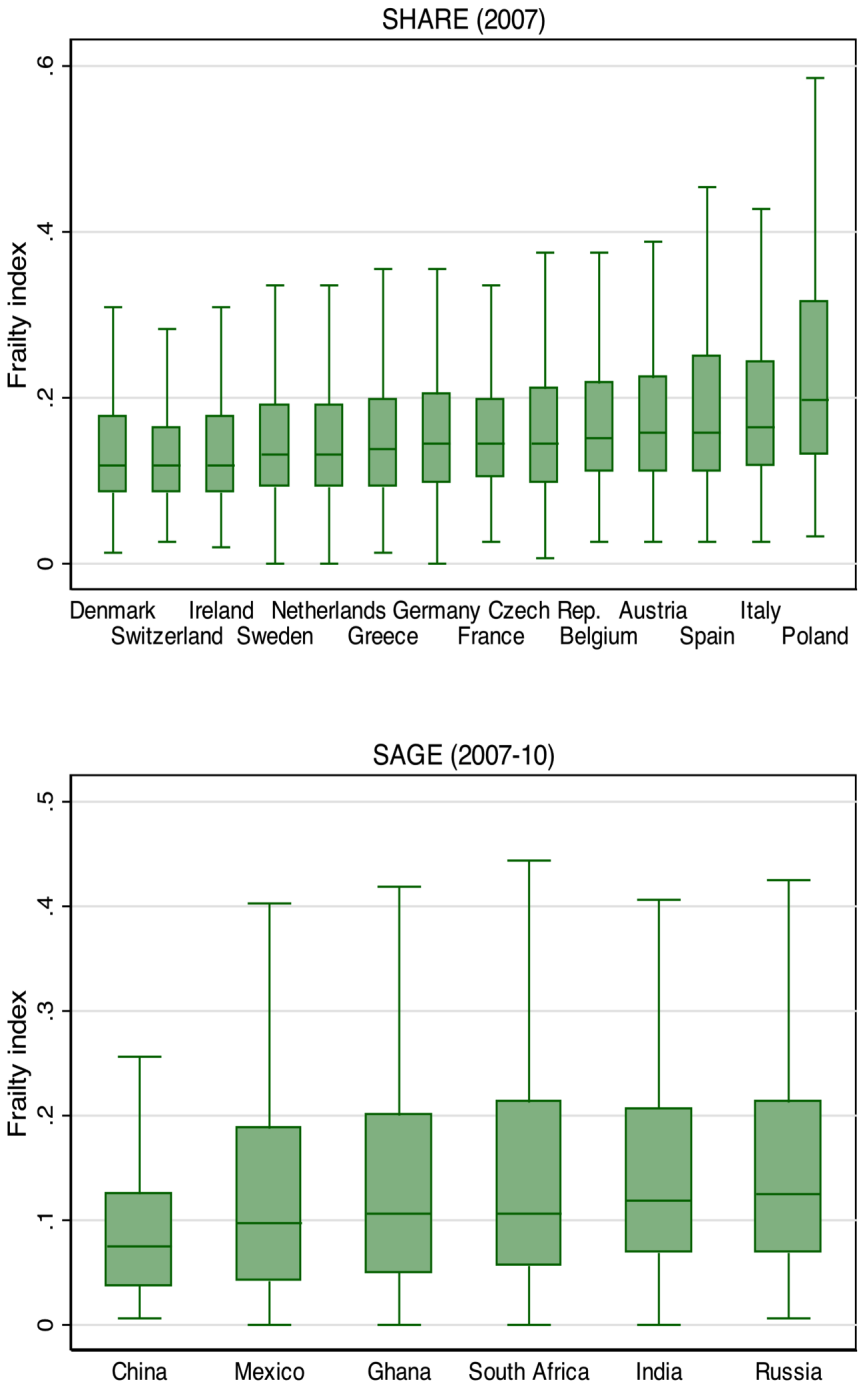
Box plots of the frailty index by country. Note: The box shows the 25^th^ to 75^th^ percentile of the frailty index. The horizontal bar inside the box shows the median value. The upper and lower bars show the upper and lower adjacent values (values within 1.5 times the IQR from the upper and lower quartile). The interquartile range (IQR) is defined as the distance between the 25^th^ and 75^th^ percentile. Outside values are omitted. Countries sorted in increasing order by the median of the frailty index.

Amongst the SHARE countries – the highest mean frailty index scores were seen in Italy, Spain and Poland, the lowest in Denmark, Switzerland and Ireland. In SAGE, the highest frailty score was seen in the Russian Federation. The lower income countries had lower frailty levels than those seen in higher income countries – with China having the lowest mean frailty scores amongst all countries (Tables 3 and 4). Figure 2 shows a box plot of the frailty index by country.

On average, women and older age groups had higher frailty levels than men and younger aged adults. The mean frailty scores demonstrated strong inverse education and income gradients, with lower levels of education and lower wealth showing higher levels of frailty. These patterns were consistent in higher and lower income countries from the two studies.

**Figure 3:**
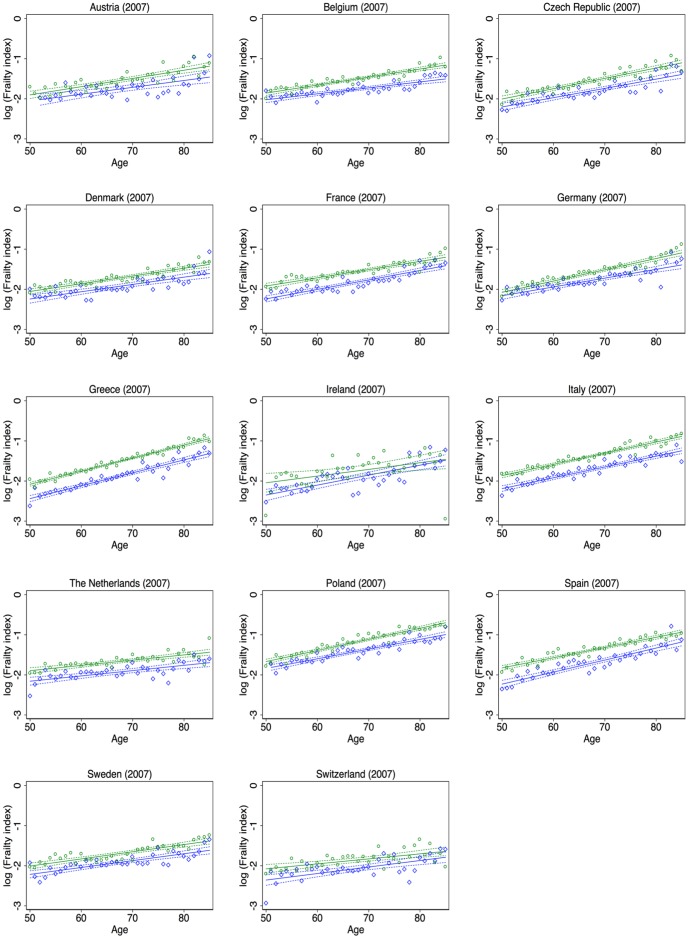
Relationship between frailty and age by country and sex, SHARE countries. Note: Green circles represent the average frailty at the respective age for females. Blue diamonds represent the average frailty at the respective age for males. Solid lines: Fit of a simple linear regression between log(Age specific frailty index) and age. Dashed lines: 95 percent confidence bands for the above mentioned regression lines. Green represents females and blue represents males.

**Figure 4:**
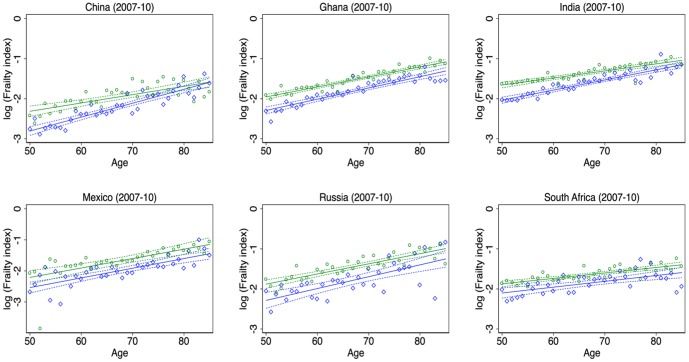
Relationship between frailty and age by country and sex, SAGE countries. Note: Green circles represent the average frailty at the respective age for females. Blue diamonds represent the average frailty at the respective age for males. Solid lines: Fit of a simple linear regression between log(Age specific frailty index) and age. Dashed lines: 95 percent confidence bands for the above mentioned regression lines. Green represents females and blue represents males.

To visualize the link between frailty and age, scatter plots of the log of the age-specific frailty index and age with a linear fit and a 95% confidence band were generated by country and sex (Figures 3 and 4). At most ages, the regression line for females is strictly above the regression line for males. In general, the slopes are rather similar for males and females within a country (Table 5). However in a few countries, such as China, India, Ireland and Switzerland, the slope is much steeper for males than for females, causing the regression lines to intersect at older ages.

**Table 5:** Relationship between frailty and age by country and sex.

	Males		Females	
	Slope	Intercept	Slope	Intercept
SHARE countries				
Austria	0.0170***	−2.906***	0.0205***	−2.927***
	(0.0098; 0.0243)	(−3.409; −2.404)	(0.0160; 0.0250)	(−3.235; −2.619)
Belgium	0.0148***	−2.764***	0.0200***	−2.855***
	(0.0114; 0.0183)	(−3.001; −2.528)	(0.0171; 0.0228)	(−3.050; −2.660)
Czech Republic	0.0231***	−3.358***	0.0257***	−3.291***
	(0.0185; 0.0278)	(−3.675; −3.042)	(0.0220; 0.0295)	(−3.545; −3.037)
Denmark	0.0181***	−3.149***	0.0193***	−3.019***
	(0.0131; 0.0232)	(−3.493; −2.805)	(0.0161; 0.0225)	(−3.237; −2.801)
France	0.0236***	−3.418***	0.0204***	−2.940***
	(0.0198; 0.0274)	(−3.675; −3.161)	(0.0172; 0.0237)	(−3.164; −2.716)
Germany	0.0220***	−3.259***	0.0281***	−3.479***
	(0.0175; 0.0264)	(−3.565; −2.954)	(0.0247; 0.0315)	(−3.712; −3.247)
Greece	0.0321***	−4.045***	0.0326***	−3.709***
	(0.0284; 0.0359)	(−4.303; −3.788)	(0.0303; 0.0349)	(−3.865; −3.553)
Ireland	0.0248***	−3.587***	0.0166***	−2.875***
	(0.0178; 0.0317)	(−4.060; −3.114)	(0.0052; 0.0281)	(−3.655; −2.095)
Italy	0.0269***	−3.535***	0.0279***	−3.260***
	(0.0235; 0.0304)	(−3.771; −3.298)	(0.0252; 0.0306)	(−3.445; −3.075)
Netherlands	0.0132***	−2.817***	0.0131***	−2.552***
	(0.0084; 0.0180)	(−3.147; −2.487)	(0.0095; 0.0168)	(−2.799; −2.306)
Poland	0.0241***	−3.045***	0.0277***	−3.052***
	(0.0206; 0.0277)	(−3.290; −2.799)	(0.0248; 0.0306)	(−3.251; −2.852)
Spain	0.0300***	−3.731***	0.0255***	−3.100***
	(0.0254; 0.0346)	(−4.047; −3.414)	(0.0226; 0.0284)	(−3.299; −2.902)
Sweden	0.0172***	−3.074***	0.0179***	−2.902***
	(0.0128; 0.0215)	(−3.372; −2.775)	(0.0142; 0.0216)	(−3.157; −2.647)
Switzerland	0.0163***	−3.169***	0.0125***	−2.703***
	(0.0098; 0.0227)	(−3.609; −2.730)	(0.0072; 0.0178)	(−3.065; −2.341)
SAGE countries				
China	0.0348***	−4.545***	0.0208***	−3.353***
	(0.0295; 0.0401)	(−4.907; −4.183)	(0.0143; 0.0273)	(−3.795; −2.912)
Ghana	0.0281***	−3.695***	0.0249***	−3.202***
	(0.0238; 0.0323)	(−3.987; −3.404)	(0.0219; 0.0280)	(−3.412; −2.993)
India	0.0253***	−3.300***	0.0176***	−2.537***
	(0.0216; 0.0290)	(−3.552; −3.047)	(0.0140; 0.0212)	(−2.780; −2.293)
Mexico	0.0312***	−4.096***	0.0303***	−3.725***
	(0.0226; 0.0397)	(−4.680; −3.512)	(0.0195; 0.0411)	(−4.463; −2.987)
Russian Federation	0.0295***	−3.763***	0.0254***	−3.148***
	(0.0195; 0.0396)	(−4.444; −3.082)	(0.0202; 0.0305)	(−3.501; −2.795)
South Africa	0.0145***	−2.828***	0.0136***	−2.557***
	(0.0082; 0.0208)	(−3.258; −2.397)	(0.0102; 0.0171)	(−2.796; −2.318)

Note:

*** denote significance at the 1 percent level. 95% confidence intervals are reported in parentheses. These are the slope coefficients and intercepts of the regression lines from Figures 3 and 4. The dependent variable is log(Age specific frailty index) and the only independent variable is age. The sample size is 36 (ages between 50 and 85).

## Discussion

Frailty is being increasingly recognized as a useful construct in clinical and other settings. While different stakeholders have used varying definitions to suit their specific purpose, there is an emerging consensus that frailty is a multidimensional phenomenon characterized by decreased reserve and diminished ability to respond to stress. Its assessment should include measures of physical performance, including gait speed and other measures of mobility, nutritional status and mental health, including cognition. However, currently there is no agreement on a single biomarker for frailty [9], [27].

A recent systematic review on the prevalence of frailty in community samples showed a consistent increase in frailty with age and that frailty was more common in women. This review also noted big differences between countries that were attributed to differences in the definition used [28]. Despite our use of a frailty index that was uniformly applied across countries we still have significant differences between countries. Our observation that higher frailty scores were seen in the SHARE countries as compared to the SAGE countries could possibly indicate a survivor bias, where social support and health systems allow people in wealthier countries to live longer despite higher levels of frailty.

Approaches to measuring frailty and constructing a frailty index have varied considerably. A recent analysis of six different approaches showed that these measures can, at best, be used to exclude frailty as their ability to predict functional decline and mortality remained poor, and their false positive rates were too high to permit major health care decisions [29]. Measuring frailty on a continuum as in our study helps in setting thresholds as fit for purpose. As the SAGE and SHARE studies will produce more data from future waves and track respondents who develop a need for long term care or die, the frailty index can be examined to determine its predictive validity including a sensitivity analysis to pick the right threshold depending on the country. Also, it would allow the examination of different trajectories that respondents may follow over time and an analysis of the factors associated with these different trajectories.

Frailty is perhaps the end result of a cascade of events from inflammatory processes to coagulative dysregulation to a range of alterations in hormones and peptides and a disruption of homeostatic mechanisms. Understanding this further in a range of settings will help identify individuals at risk of becoming frail and refine interventions for prevention and treatment that will likely range from nutritional therapies to improving physical activity [30].
